# Magnesium metabolism: A potential breakthrough in osteoporosis intervention

**DOI:** 10.1016/j.isci.2025.114437

**Published:** 2025-12-15

**Authors:** Wei Xiong, Yuxiang Zhou, Yilin Yan, Yuyi Li, Jingkun Li, Yifeng Yuan, Xiaolin Shi, Kang Liu

**Affiliations:** 1 The Second School of Clinical Medicine, Zhejiang Chinese Medical University, Hangzhou, Zhejiang, China; 2 Rehabilitation Department, Zhoushan Guanghua Hospital, Zhoushan, Zhejiang, China; 3 Xinyu Traditional Chinese Medicine Hospital, Xinyu, Jiangxi, China; 4 The Second Afffliated Hospital of Zhejiang Chinese Medical University, Hangzhou, Zhejiang, China

**Keywords:** Therapeutics, Human metabolism

## Abstract

Osteoporosis (OP), a multifactorial systemic skeletal disease, is characterized by significant reductions in bone density and quality, coupled with progressive deterioration of bone microarchitecture, and poses a significant health challenge. Conventional therapies, while partially effective, have limitations. Recent studies highlight the crucial role of magnesium (Mg) metabolism in bone health. Mg regulated bone homeostasis by promoting osteoblasts (OBs) differentiation and suppressing osteoclasts (OCs) activity. They also influence intracellular metabolism and hormonal functions. Mg deficiency can disrupt calcium (Ca) homeostasis, exacerbating bone resorption. Novel interventions, including Mg supplementation, Mg-based implants, offer promising approaches. This review synthesizes evidence on Mg’s impact on OP pathogenesis and evaluates these therapeutic strategies, aiming to guide future research and clinical applications. It underscores Mg metabolic balance as a critical target for OP management.

## Graphical abstract

**Figure:**
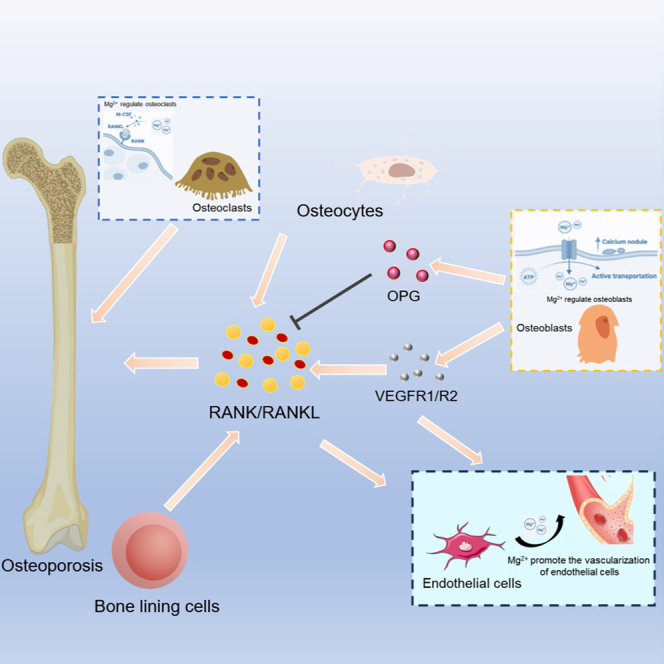

## Introduction

Bone formation is a highly dynamic and finely regulated physiological process that centers on the intricate interactions between osteoblasts (OBs) and osteoclasts (OCs).^1^^,^^2^ Concurrently, other functional cells—including osteocytes (OCY) and endothelial cells (ECs)—play critical regulatory roles in bone formation.^3^^,^^4^ OCs mediate bone resorption by clearing aged bone matrix, thereby creating space for new bone deposition. OBs synthesize and mineralize nascent bone matrix, thereby facilitating bone tissue remodeling and maintaining structural integrity. OCY, embedded within mineralized bone matrix, form interconnected networks primarily responsible for regulating bone microenvironmental homeostasis.^5^ ECs drive bone vascularization by constructing microvascular networks that deliver oxygen, nutrients, and growth factors to OCs.^6^ When bone metabolic equilibrium is disrupted—for instance, due to abnormally enhanced OCs activity coupled with impaired OBs function and reduced activity—bone mineral density declines and bone strength is compromised, thereby significantly elevating the risk of metabolic bone disorders such as OP.^7^

**Figure 1:**
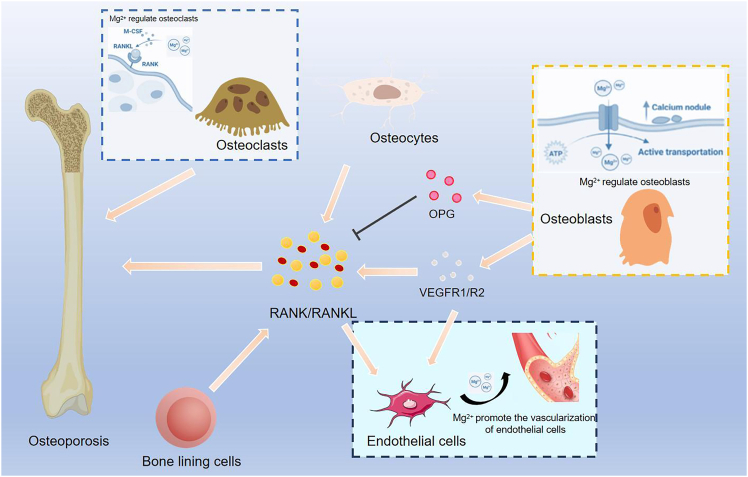
RANKL/RANK/OPG axis imbalance: the core molecular mechanism of osteoporosis The RANKL/RANK/OPG axis is a pivotal regulatory system for bone homeostasis, primarily controlled by osteoblasts which express RANKL to promote osteoclast differentiation and OPG to inhibit it; the core mechanism involves RANKL binding to RANK on osteoclast precursors to activate differentiation, while OPG acts as a decoy receptor that competitively blocks this interaction. Furthermore, osteocytes serve as a crucial source of RANKL, bone lining cells express it during active remodeling to maintain metabolic balance, and there is a link to angiogenesis where VEGF upregulates RANK on endothelial cells, enhancing their pro-angiogenic response to RANKL. An imbalance in this axis is a key factor leading to osteoporosis.

Magnesium (Mg), an essential nutrient vital for skeletal development and mineralization, plays a significant role in the pathogenesis of OP,.^8^ Mg deficiency can indirectly impair bone structure by affecting two key regulators of calcium homeostasis: parathyroid hormone (PTH) and vitamin D.^9^ In human OBs, PTH modulates OC formation by regulating the expression of receptor activator of nuclear factor κB (RANK), receptor activator of nuclear factor κB ligand (RANKL), and osteoprotegerin (OPG). Additionally, Mg may influence vitamin D3-mediated bone remodeling, which typically coordinates the activation balance between OBs and OCs.^10^ Dysregulated activation of OCs ultimately results in excessive bone resorption.^11^ As show in Figure 1, the RANK/RANKL/OPG axis has been identified as a central molecular mechanism underlying OP pathogenesis. Mg participates in OP development by modulating this axis through its influence on parathyroid hormone and vitamin D levels. Various pathological factors can disrupt this axis and enhance OC function, ultimately resulting in bone loss and microarchitectural deterioration.

Building upon the multifaceted regulatory mechanisms of magnesium ions (Mg^2+^) in bone metabolism, novel OP intervention strategies have emerged, including Mg supplementation therapy to correct deficiency states and Mg implants for OP-related fracture treatment.^12^ Beyond these applications, Mg^2+^ demonstrates unique capabilities in locally modulating the osteoimmune microenvironment, promoting angiogenesis and neuroregeneration, thereby serving as a potent synergistic agent to enhance anti-osteoporotic efficacy.^13^ Of particular clinical significance, Mg^2+^ deficiency can induce inflammatory cytokine infiltration into the bone metabolic microenvironment, aggravating oxidative stress and establishing a vicious “inflammation-bone loss” cycle.^14^ These compelling findings collectively establish Mg^2+^ metabolic imbalance as a critical driver in OP development and progression.

This review systematically synthesizes current evidence regarding Mg’s impact on OP pathogenesis and evaluates potential therapeutic strategies, aiming to provide clinical researchers with novel directions to advance the development and application of Mg-based interventions in OP management.

## The physiological function of Mg^2+^ and its regulation in bone homeostasis

**Figure 2:**
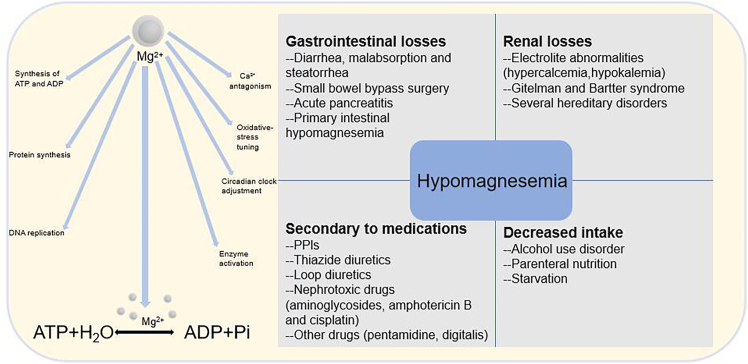
Schematic diagram of physiological functions of Mg^2+^ and common causes of hypomagnesemia This schematic diagram illustrates that Mg^2+^ are essential cofactors in fundamental physiological processes such as energy metabolism, notably in the hydrolysis of ATP (ATP + H_2_O → ADP + Pi). Hypomagnesemia, or low serum magnesium, can arise from a variety of causes, which are categorized into (1) gastrointestinal losses (e.g., diarrhea, malabsorption, steatorrhea, small bowel bypass surgery, acute pancreatitis, and primary intestinal hypomagnesemia); (2) renal losses due to electrolyte abnormalities (like hypercalcemia and hypokalemia) or hereditary disorders such as Gitelman and Bartter syndromes; (3) medications including proton pump inhibitors (PPIs), thiazide and loop diuretics, nephrotoxic drugs (e.g., aminoglycosides, amphotericin B, and cisplatin), and others like pentamidine and digitalis; and (4) decreased intake associated with alcohol use disorder, parenteral nutrition, and starvation.

Mg^2+^ is the body’s second most abundant cation, playing a critical role in maintaining physiological functions and bone homeostasis.^15^ As show in Figure 2, they participate in numerous intracellular metabolic processes, activate over 300 enzymes linked to carbohydrate, nucleic acid, and protein metabolism, and act as membrane stabilizers to maintain electrolyte balance and cellular integrity.^16^ Mg^2+^ also plays a key role in muscle contraction, nerve excitability, and the release of hormones and neurotransmitters.^17^ In terms of skeletal health, Mg^2+^ is indispensable for bone development and mineralization.

It primarily stabilizes calcium phosphate crystals and enhances bone matrix mechanical properties by incorporating into hydroxyapatite crystals. Animal studies show that Mg^2+^ deficiency reduces bone density and mechanical strength, while clinical data indicate that hypomagnesemia is associated with lower hip bone density and higher fracture risk.^18^ Mg^2+^ regulates bone metabolism by modulating the RANKL/RANK/OPG pathway, promoting OB differentiation and bone mineralization, and suppressing OC formation and activity.^19^ This pathway is central to bone metabolism and immune function. OB-secreted RANKL binds to the RANK receptor on OC precursors, promoting their maturation into OCs in the presence of macrophage colony stimulating factor. OPG, a soluble decoy receptor for RANKL produced by OBs, inhibits OCs formation by blocking RANKL-RANK interactions. When RANKL binds to RANK, it triggers a signaling cascade involving TNF receptor-associated factor 6, recruits and activates inhibitor of kappa B kinase (IκB). IκB degradation, nuclear factor kappa-B (NF-κB) activation, and nuclear translocation, lead to gene expression and inflammatory responses that enhance bone resorption and reduce bone mass.

Mg^2+^ also indirectly affects bone homeostasis by regulating Ca and phosphate metabolism. Mg^2+^ deficiency reduces PTH secretion and 1,25(OH)_2_D_3_ activity, impairing intestinal calcium ions (Ca^2+^) absorption and skeletal Ca^2+^ mobilization.^20^ PTH increases OCs’ activity and indirectly activates them via OBs’ metabolic influences. In OBs, the interplay of RANKL and OPG maintains bone stability. Research shows that PTH administration decreases OPG mRNA expression in rat bones, likely via protein kinase A (PKA)-CREB-AP-1 pathway activation.^21^ Bone metabolism is intricately connected to the processes of bone remodeling and repair, with bone angiogenesis playing a central role in these mechanisms.^19^ Emerging research indicates that Mg^2+^ contributes not only to the promotion of blood vessel formation in bone but also actively influences the regulation of bone metabolic activities. During angiogenesis, the transformation of ECs into tip cells is a critical step, as these tip cells guide the direction and structural development of newly formed vascular branches.^22^ Studies have demonstrated that Mg^2+^ enhances the differentiation of tip cells via the combined action of the vascular endothelial growth factor (VEGF)A-VEGFR2 and Notch1 signaling pathways. This process facilitates the nuclear translocation of YAP, which in turn boosts the migration of tip cells and the formation of filopodia, while simultaneously encouraging the proliferation of stalk cells—ultimately supporting the development and maturation of a functional vascular network.^23^ In summary, Mg^2+^ plays a crucial role in regulating bone homeostasis and metabolism. Therefore, we believe that the optimal efficacy in protecting bone health and delaying or preventing the development of OP can be achieved only through the combined application of multiple bone nutrients (such as magnesium, calcium, vitamin D, protein, and potentially more) in interventions aimed at improving bone health.

## Association between abnormal Mg^2+^ metabolism and pathogenesis of OP

**Figure 3:**
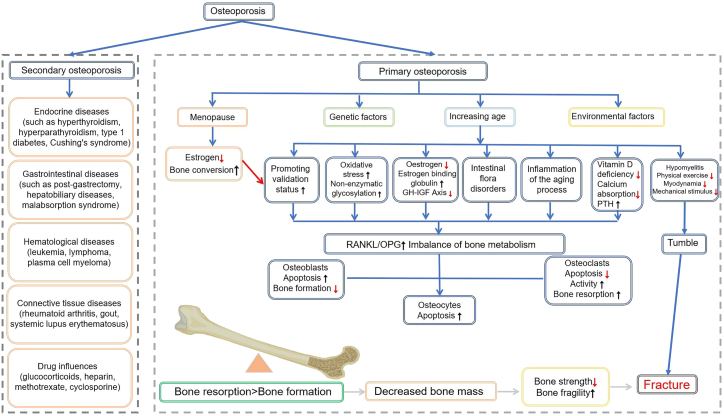
Pathophysiology and classification of OP OP is primarily classified into primary and secondary types: primary OP, including postmenopausal, senile, and idiopathic forms, is linked to estrogen deficiency, age-related factors, or unknown causes, whereas secondary OP results from underlying conditions or medications, such as endocrine, gastrointestinal, hematological, and connective tissue diseases. The pathogenesis is driven by core mechanisms like an imbalance in the RANKL/OPG axis, oxidative stress, gut microbiota dysbiosis, and vitamin D deficiency. These dysregulations lead to heightened OC activity, causing bone resorption to outpace bone formation, which ultimately results in reduced bone mass, increased bone fragility, and a significantly elevated fracture risk.

OP can be categorized into primary and secondary types.^24^ Primary OP includes postmenopausal OP (Type I), senile OP (Type II), and idiopathic OP. Postmenopausal OP is typically linked to the sharp decline in estrogen levels in women after menopause. As show in Figure 3, senile OP is associated with age-related bone metabolic imbalances in the elderly. The etiology of idiopathic OP, however, remains unclear.^25^ In addition, various other diseases can lead to OP. These include endocrine disorders (such as hyperthyroidism and Cushing’s syndrome), connective tissue diseases (such as rheumatoid arthritis and systemic lupus erythematosus), chronic kidney disease, gastrointestinal disorders (such as malabsorption syndrome), and hematological diseases (such as leukemia and lymphoma).^26^^,^^27^ These conditions disrupt bone metabolism through various mechanisms, such as interfering with the metabolic balance of Ca, phosphorus, and Mg, affecting vitamin D activation, inhibiting OB function, or activating OCs. Consequently, they disrupt the bone remodeling process and ultimately lead to OP.

Mg is essential for bone development and mineralization, promoting bone health by enhancing phosphatase activity. However, inadequate dietary Mg intake can result in decreased bone density. Animal studies indicate that Mg^2+^ deficiency contributes to OP development by reducing bone density and altering tissue levels of PTH and 1,25(OH)_2_D, thereby inhibiting calcium absorption and causing hypocalcemia.^28^ Long-term clinical studies have demonstrated that hypomagnesemia significantly suppresses PTH synthesis, impairing the function of related organs.^29^ For example, research by Ohya et al. has confirmed a significant correlation between serum Mg^2+^ levels and intact PTH (iPTH) levels. Additionally, research by Cheung et al. indicates that combined Mg^2+^ and vitamin D supplementation may be more effective in increasing serum 1,25(OH)_2_D_3_ concentrations than vitamin D alone.^30^ As Mg is a necessary cofactor for numerous enzymes, its deficiency may increase resistance to PTH, thereby affecting enzyme activity. Notably, hypomagnesemia can also trigger inflammatory responses, further exacerbating bone loss. Studies also suggest that Mg^2+^ deficiency may reduce the volume of bone marrow vessels, potentially leading to neurogenic OP, particularly in elderly individuals.

### The effect of Mg metabolism on OBs in the pathogenesis of OP

Mg^2+^ significantly impact OBs differentiation and function via complex, multi-pathway mechanisms. Extracellular Mg^2+^ interacts with transient receptor potential melanoma 7 (TRPM7), influencing platelet-derived growth factor (PDGF)-induced human OB proliferation and migration.^31^ Mg^2+^ also regulates PTH secretion, akin to Ca.^32^ When serum Mg^2+^ rises, it binds to Ca-sensing receptors on parathyroid cells, increasing intracellular Ca and reducing PTH secretion. Conversely, low serum Mg^2+^ elevates PTH levels. PTH promotes bone formation by activating OBs, inducing insulin-like growth factor (IGF)-1 production, and potentially inhibiting sclerostin (SOST). Through enzymes like PKA, PTH drives OB proliferation.^33^ Research in animal models and human trials shows PTH administration significantly promotes OB proliferation and accelerates bone formation. PTH also enhances mineralized matrix deposition by regulating OB precursor proliferation.^34^

In OP pathogenesis, magnesium metabolism plays a crucial role in regulating OBs function. PTH primarily reduces OBs’ apoptosis rather than promoting pre-OB proliferation. PTH signaling affects the transcription factor Runx2, which is closely related to OBs differentiation and function.^35^ Beyond Runx2, multiple genes like Tmem119, IGF-1, FGF2, PTH-related protein, and MMP13 contribute to the anti-apoptotic effects of iPTH therapy.^36^^,^^37^ iPTH also plays a significant role in OB differentiation and function through the WNT signaling pathway.^38^ However, WNT signaling differentially affects OBs at various differentiation stages. In pre-OBs, WNT signaling stimulates cell proliferation, while in mature OBs, it increases OPG levels, slowing bone resorption.^39^ PTH also promotes bone growth via the Notch signaling pathway by upregulating Jag1 and other components like Notch2 and Dll1. Notch1 and Notch2 have inhibitory effects on bone cell function, but their signaling activation can promote bone cell differentiation and absorption.^40^

Mg deficiency adversely affects OBs and bone health. Hypomagnesemia often correlates with low serum levels of the active vitamin D metabolite 1,25(OH)_2_D_3_. This may result from low PTH levels or renal PTH resistance, as PTH is a key physiological regulator of 1,25(OH)_2_-VD synthesis.^41^ Magnesium is essential for this synthesis. Prior studies show 1a,25(OH)_2_D_3_ directly impacts OB survival, with effects varying by treatment time, dose, source, and environment. It also affects OB proliferation and differentiation.^42^ Both extracellular and intracellular environments significantly influence the effects of 1a,25(OH)_2_D_3_.^43^ A key intracellular pathway is the WNT signaling pathway. Standard WNT signaling is vital for bone formation. Lipoprotein receptor-related proteins 5 and 6 (LRP5/6) facilitate the secretion of WNT family proteins and bind to membrane receptors on bone cells. 1a,25(OH)_2_D_3_ promotes the binding of vitamin D receptor to LRP5 sites, making VD_3_ a crucial factor in bone cell differentiation and bone formation due to its influence on WNT signaling.^44^

In summary, Mg regulates OBs function primarily through PTH and vitamin D-mediated signaling pathways (e.g., WNT and Notch). The evidence for fundamental molecular mechanisms is strong but largely derived from *in vitro* and animal studies. Direct clinical evidence linking Mg levels to bone formation outcomes in humans remains limited, relying heavily on indirect associations. The precise hierarchy of these pathways requires further clarification.

### The effect of Mg metabolism on OCs in the pathogenesis of OP

In the pathogenesis of OP, augmented OCs activity is pivotal. As a key metabolic element, Mg^2+^ markedly inhibits OCs’ activity.^45^ Both preclinical and clinical studies indicate that Mg^2+^ boosts OBs’ activity and inhibits OCs’ formation and function through multiple pathways.^46^ Specifically, Mg^2+^ regulates mature OC activity, migration, and precursor cell activity, curbing OCs generation.^47^ Moreover, by modulating the expression of OPG, RANKL and NF-κB, Mg^2+^ influences OCs formation, playing a crucial role in bone metabolism balance, and holding promise for OP prevention.^48^

OP also correlates with abnormal OCs precursor cell differentiation. Mg^2+^ plays a significant role in regulating this process.^49^ Derived from mononuclear macrophage precursors, OCs formation can be hindered by Mg^2+^.^50^ For instance, the alkaline microenvironment from degrading Mg-coated titanium materials inhibits the differentiation of bone marrow macrophages into OCs.^51^ While some studies show no significant effect of varying Mg^2+^ concentrations on precursor cell proliferation, high Mg^2+^ levels reduce their metabolic activity and ability to fuse into multinucleated OC-like cells, possibly due to inhibited cell fusion. However, the mechanism underlying Mg^2+^’s impact on precursor cell differentiation requires further study.

In the context of OP, Mg metabolism is vital for regulating bone metabolic microenvironments and OCs activity. The alkaline microenvironment generated by degrading Mg-based implants suppresses OCs activity. OC-mediated bone resorption activity is pH dependent, intensifying in acidosis and diminishing in alkaline conditions.^52^ The local alkaline environment from Mg^2+^ release inhibits the expression of OCs-associated enzymes and factors, such as carbonic anhydrase II, vacuolar H^+^-ATPase, cathepsin K, and bone resorption-related factors (TNF, NFATc1, and TRAP), thereby inhibiting OC activity.^53^ Additionally, Mg^2+^ suppresses TNF-α formation and release, affecting OC adhesion and regulating bone resorption.^54^ Despite this, the specific mechanisms of Mg metabolism’s regulation of OCs activity remain largely unknown. Further research could facilitate the development of more effective OP therapies.

In conclusion, Mg exerts a net inhibitory effect on OCs, supported by strong *in vitro* evidence and observations from Mg-based biomaterials. However, evidence is inconsistent regarding its direct impact on precursor cells, and on the specific intracellular mechanisms there remains a significant knowledge gap, necessitating further investigation.

### Effects of Mg metabolism on OCY in the pathogenesis of OP

OCY, hailed as master mechanosensory cells, are vital for bone remodeling. PTH inhibits SOST synthesis in OCY, boosting bone formation.^55^ Yet, Mg^2+^ deficiency can curb PTH secretion, reducing skeletal and renal hormone sensitivity. This inhibition affects bone remodeling and vitamin D production in the kidneys, processes tied to OP.^55^ OCY also modulate bone metabolism by regulating RANKL expression, a key factor in osteoclastogenesis that is upregulated by PTH. Mg^2+^ supplementation can influence PTH secretion by tweaking RANKL levels in OCY, thus steering bone remodeling.^56^

Moreover, Mg^2+^ deficiency disrupts bone metabolism by impacting the Ca regulator in bone—1,25(OH)_2_D_3_. Research shows OCY activity is linked to blood phosphate and 1,25(OH)_2_D_3_ levels. Pereira’s team found that active vitamin D sterols can reverse bone metabolic disorders caused by magnesium deficiency.^57^ In chronic kidney disease patients, these sterols promote early OCY maturation, increase late-stage OCY numbers, and boost OCY turnover. Vitamin D may also regulate RANKL/OPG expression, making it a key player in coupling OBs, OCs, and OCY, and playing a major role in bone metabolism.

Altogether, Mg influences OCs indirectly by modulating PTH and vitamin D, which affect key OC functions such as SOST secretion. The current evidence is largely indirect and extrapolated, with a clear need for studies directly linking Mg levels to OCY signaling *in vivo*.

### Effects of Mg metabolism on ECs in the pathogenesis of OP

Bone angiogenesis is closely linked to bone metabolism, remodeling, and repair. Studies show Mg^2+^ promotes bone angiogenesis, influencing OP regulation.^58^ During angiogenesis, EC differentiating into tip cells is key, as it directs new vascular sprout growth.^59^ Research indicates Mg^2+^ can promote this differentiation via VEGFA-VEGFR2/Notch1 signaling crosstalk. It also induces YAP nuclear translocation, boosting tip cell migration and filopodia formation, and stalk cell proliferation, thus maturing the vascular network. Applying Mg-loaded hydrogels to bone injuries enriches the skeletal vascular network, accelerating bone formation.

OP is prevalent among the elderly, characterized by reduced vascularization in bone tissue, disrupted bioelectric signals, ion imbalances, and decreased stem cell differentiation, leading to bone defects that are difficult to repair. The WU team innovatively used Mg^2+^-containing black phosphorus (BP) conductive hydrogel, employing a synergistic treatment strategy to successfully reverse these pathological imbalances, restore Mg^2+^ homeostasis, reconstruct physiological signals, and promote vascular regeneration at OP bone defects, thereby achieving bone defect repair.^60^ The team also discovered that Mg^2+^ can activate the PI3K-AKT-eNOS signaling pathway, enhancing the expression levels of VEGF and its receptor (VEGFR2), significantly boosting the angiogenic capacity of vascular ECs in an aging state. Additionally, this hydrogel helps normalize Ca^2+^ influx, increasing the accumulation of osteogenic transcription factors in the cell nucleus, thus promoting the differentiation of senescent stem cells into OBs.

In the skeletal system, VEGF, activated by hypoxia inducible factor-1α (HIF-1α) nuclear entry, is key in angiogenesis-osteogenesis coupling.^61^^,^^62^ Mg^2+^ also promotes PDGF subunit B (PDGF-BB) secretion by MC3T3-E1 cells, inducing H-type vascular EC differentiation and activating angiogenesis-osteogenesis coupling.^63^ Additionally, Qin et al. discovered Mg^2+^ promotes the angiogenic differentiation of bone marrow mesenchymal stem cells (BMSCs) by activating the Notch signaling pathway, upregulating Hes1, Hes5, Hif-α, and eNOS.^64^

Cumulatively, Mg promotes bone angiogenesis by activating key pathways (e.g., VEGFR2/Notch and PI3K/AKT) in ECs. The evidence is compelling in the context of bone repair models using Mg-containing materials, but its direct role in systemic osteoporosis-related vascular decline is less established and represents a promising future direction.

### Effects of Mg metabolism on other cells in the pathogenesis of OP

In addition to the mechanisms described above, the therapeutic role of magnesium in OP extends far beyond the traditional notions of “promoting calcium absorption” or “constituting bone minerals.” A growing body of research has revealed that one of its core modes of action is the modulation of two pivotal players in the bone-marrow micro-environment: BMSCs and immune cells.

As the principal “builders” of bone, BMSCs are multipotent stem cells resident in the marrow that serve as common progenitors for both OBs (which form bone) and adipocytes (which occupy marrow space without contributing to bone accrual). The health of skeletal metabolism therefore hinges largely on the lineage “fate decision” of BMSCs—whether they differentiate into OBs or adipocytes. Optimal extracellular magnesium concentrations have been shown to activate canonical osteo-inductive signaling cascades such as Wnt/β-catenin and BMP/Smad.^65^ Once triggered, these pathways up-regulate OB-specific transcription factors (e.g., Runx2 and Osterix), channeling BMSCs toward an osteoblastic phenotype. Concomitantly, magnesium-treated BMSCs exhibit heightened alkaline phosphatase (ALP) activity, augmented type-I collagen (COL-I) deposition, and more robust mineralized-nodule formation—hallmarks of mature osteoblastic function.^66^

Importantly, while magnesium steers BMSCs toward osteogenesis it simultaneously suppresses adipogenic commitment by down-regulating key adipogenic transcription factors such as PPARγ and C/EBPα. By limiting marrow adiposity, magnesium helps preserve the hematopoietic and osteogenic niches, thereby counteracting a well-recognized driver of osteoporotic change.^67^

On the other hand, bone is not an isolated organ; its metabolism is continuously fine-tuned by the immune system—a discipline now recognized as osteoimmunology.^68^ Magnesium functions as a critical “mediator” within this crosstalk. First, it can dictate macrophage polarization: ample magnesium steers monocyte-derived macrophages toward the M2 phenotype, thereby shifting the marrow milieu from a pro-osteoclastic, inflammatory state to a pro-osteogenic, reparative one.^69^ Second, magnesium acts as a physiological “brake” on T cell activation. When magnesium is scarce, T cells hyper-activate, bias toward Th17 differentiation, and amplify osteoclastic bone destruction. Conversely, sufficient magnesium preserves both the suppressive capacity and the pool size of regulatory T (Treg) cells, sustaining immune tolerance that favors balanced skeletal remodeling.^70^

## Intervention strategies for OP based on Mg metabolism regulation

Mg is crucial for regulating the musculoskeletal system’s normal physiological activities. Recently, more studies have focused on Mg intake’s impact on bone and muscle health. In 2009, a team of Italian medical experts published a review article exploring the causal link between dietary Mg^2+^ intake and normal bone maintenance.^71^ By systematically summarizing 28 studies published before 2009, the review found that low serum Mg^2+^ levels are associated with OP. It highlighted that over one-third of the subjects, primarily menopausal women, suffered from both hypomagnesemia and OP. Dietary surveys indicated that approximately 20% of the subjects had Mg^2+^ intake below the recommended level. Currently, the main dietary forms of Mg are citrate, carbonate, and oxide, with doses ranging from 250 to 1,800 mg. This intake level is considered beneficial for increasing bone mineral density and reducing fracture risk.^72^

A clinical case-control study conducted in Israel revealed that among female patients, 71% experienced a significant increase in bone density after Mg^2+^ supplementation, and some OP patients saw their bone loss halted.^73^ For menopausal women, the combination of Mg^2+^ supplements with other elements showed better results in preventing OP. A previous study found that compared to patients with primary postmenopausal osteoporosis (PPMO) taking only 500 mg of Ca citrate, PPMO patients taking a combined supplement of 500 mg Ca citrate and 200 mg Mg oxide had significantly higher average calcaneal bone density.^74^ For OP patients, the recommended Ca-Mg intake ratio is between 2.2 and 3.2. Notably, Mg^2+^ deficiency leads to a higher fracture risk in women (62%) than in men (53%), likely due to the stronger correlation between women’s bone density and Mg^2+^ intake.

On the other hand, a study by a Britain research team based on the NHANES database showed a positive correlation between dietary Mg^2+^ intake and the appendicular skeletal muscle mass index.^75^ However, this association was not found when using Mg^2+^ supplements, underscoring the importance and uniqueness of Mg^2+^ from dietary sources. In addition, Mg^2+^ can enhance muscle performance in the musculoskeletal system. This is possibly related to Mg^2+^’s role in promoting muscle protein synthesis, accelerating energy metabolism, and increasing glucose availability in muscles and blood.^76^ Mg^2+^ deficiency can increase the production of pro-inflammatory cytokines and exacerbate oxidative stress responses, thereby intensifying muscle tissue inflammation. Inflammation is one of the key factors in the development of myasthenia. Several studies have shown that Mg^2+^-deficient experimental animals exhibit systemic inflammatory responses, with significantly elevated levels of inflammatory markers in pathological tests. In contrast, a higher Mg^2+^ intake can lower serum Ca-reactive protein levels.^77^^,^^78^

Mg^2+^’s active functions offer new strategies for treating OP-related diseases. OP fractures, known for slow healing, pose a clinical challenge. While the bone immune microenvironment is crucial for fracture healing, direct evidence is still insufficient.^79^ Single-cell studies show that Mg-based implant materials can increase immature neutrophils and enhance lymphocytes’ and macrophages’ anti-inflammatory capacity. Released Mg^2+^ activate the TRPM7/S100A4 pathway in macrophages, promoting osteovascularogenesis and accelerating fracture healing.^80^ Bone defects are a major challenge in skeletal repair for OP patients. Cao’s team developed multifunctional microsphere scaffolds (GMA MSs) based on a gelatin matrix, combining alendronate (ALN) and Mg^2+^-loaded microspheres. Experiments show GMA MSs can eliminate reactive oxygen species in inflammatory cells, induce macrophage polarization, and exert anti-inflammatory effects by inhibiting the PI3K/Akt and NF-κB pathways.^81^ Moreover, ALN in GMA MSs suppresses OCs activity, while Mg^2+^ induces osteogenic differentiation, and their combined action restores bone metabolic balance.

## Current challenges and future research directions

OP interventions targeting Mg metabolism show promise but face several challenges. The dosage of Mg^2+^ is critical. They’re only effective within a specific range, and high doses can cause toxicity, long-term tissue damage, and cellular dysfunction.^82^ Excess Mg^2+^ also reduce bone stiffness and strength by competing with Ca^2+^, forming insoluble salts with pyrophosphate, and inhibiting hydroxyapatite crystal formation. These salts are not enzyme degradable.^83^^,^^84^ Additionally, Mg^2+^ antagonize Ca^2+^, and high-dose Mg alters the Ca-Mg ratio in bone metabolism microenvironments, causing cellular dysfunction and worsening OP.^85^ Mg^2+^ deficiency is equally harmful, causing inflammation, activating white blood cells and macrophages, releasing inflammatory cytokines and acute-phase proteins, and generating excessive free radicals. The inflammation mechanism involves Ca^2+^-induced phagocyte activation, Ca^2+^ channel and N-methyl-D-aspartic acid receptor activation, neurotransmitter release, membrane oxidation, and NF-κB activation. These pro-inflammatory cytokines play a key role in normal bone remodeling and the pathogenesis of perimenopausal and senile OP.

To address these challenges, future research should focus on Mg^2+^ release kinetics. Advanced computational modeling and *in vitro* simulations can predict their cytotoxicity thresholds, aiding in designing safe and effective Mg^2+^ supplements. Current animal models for testing these supplements have limitations. Small animal models, while common for assessing drug toxicity, often fail to fully replicate human bone repair processes, especially the complex osteoporotic microenvironments caused by Mg^2+^ metabolic imbalances. Moreover, Mg-based metal drug carriers may show significant differences between animal models and clinical outcomes. For instance, rabbits differ from humans in bone density and tissue composition, and mice heal fractures faster, making them less suitable for simulating human bone repair. Existing animal models also rarely account for bone regeneration in patients with common bone diseases like OP and diabetes, limiting the clinical application of drug research results.^86^ Therefore, developing more appropriate animal models and promoting the clinical translation of Mg^2+^ supplements and carriers is urgently needed.

Most Mg in the human body is stored in bones and teeth (about 60%) and within cells (about 40%), with less than 1% in the blood.^87^ However, serum Mg^2+^ concentration, typically ranging from 0.75 to 0.95 mmol/L, is the common clinical measure for body Mg^2+^ levels. This method doesn’t accurately reflect total body Mg^2+^.^88^ Research shows that even with severe Mg^2+^ deficiency in bones and muscles, blood Mg^2+^ may remain normal, making this subclinical deficiency undetectable by serum testing.^89^ Thus, there’s an urgent need for a new and reliable method to assess Mg^2+^ levels, considering both serum concentration and total skeletal Mg^2+^, to better inform OP diagnosis and treatment.

Finally, while Mg^2+^ supplementation alone shows modest benefits, its integration into multi-nutrient or biomaterial-based strategies remains underexplored. For instance, co-delivery of Mg^2+^ with vitamin D, vitamin K2, and calcium may synergistically enhance OB mineralization while suppressing OC overactivation, but optimal ratios and temporal sequencing remain undefined. Similarly, Mg^2+^-loaded hydrogels or scaffolds that simultaneously modulate immune polarization (e.g., M1→M2 shift) and enhance angiogenesis-osteogenesis coupling represent a promising but under-optimized avenue. Future research should employ multi-omics and AI-driven modeling to predict nutrient-cell-matrix interactions and design personalized Mg^2+^-based combinatorial therapies.

## Conclusion

Mg metabolism emerges as a pivotal factor in the pathogenesis and management of OP. The multifaceted roles of Mg^2+^ in regulating bone homeostasis, including its influence on OBs, OCs, OCY, and ECs, underscore its significance in maintaining skeletal health. Accumulating evidence supports the potential of Mg-based interventions, such as supplementation and implant materials, in mitigating OP and promoting fracture healing. However, several challenges remain. The precise dosage of Mg^2+^ is crucial, as both deficiency and excess can have detrimental effects on bone health. Moreover, the development of accurate methods for assessing total body Mg^2+^ levels and the creation of more representative animal models for clinical translation are urgently needed. Future research should focus on optimizing Mg^2+^ release kinetics and exploring its mechanisms of action in bone metabolism.

## Acknowledgments

The authors declare financial support was received for the research, authorship, and/or publication of this article. This work was supported by “Pioneer” and “Leading Goose” R&D Program of Zhejiang (2025C02193). 10.13039/501100004731Zhejiang Provincial Natural Science Foundation (LZ22H270002, ZCLQ24H2701).

## Author contributions

Conceptualization, W.X. and Y.Z.; methodology, W.X. and Y. Yan; investigation, Y.L. and Y. Yuan; resources, J.L.; writing – original draft preparation, W.X.; writing – review and editing, W.X. and Y.Z.; supervision, X.S.; project administration, K.L.; funding acquisition, X.S. All authors have read and agreed to the published version of the manuscript.

## Declaration of interests

The authors declare no conflict of interest.
